# Automated Caries Detection Under Dental Restorations and Braces Using Deep Learning

**DOI:** 10.3390/bioengineering12050533

**Published:** 2025-05-15

**Authors:** Yi-Cheng Mao, Yuan-Jin Lin, Jen-Peng Hu, Zi-Yu Liu, Shih-Lun Chen, Chiung-An Chen, Tsung-Yi Chen, Kuo-Chen Li, Liang-Hung Wang, Wei-Chen Tu, Patricia Angela R. Abu

**Affiliations:** 1 Department of Operative Dentistry, Taoyuan Chang Gang Memorial Hospital, Taoyuan City 33305, Taiwan; louiszzzzz@cgmh.org.tw; 2 Department of Program on Semiconductor Manufacturing Technology, Academy of Innovative Semiconductor and Sustainable Manufacturing, National Cheng Kung University, Tainan City 701401, Taiwan; m28121562@gs.ncku.edu.tw; 3 Department of Electronic Engineering, Chung Yuan Christian University, Taoyuan City 32023, Taiwan; s11026211@cycu.edu.tw (J.-P.H.); s11026214@cycu.edu.tw (Z.-Y.L.); chrischen@cycu.edu.tw (S.-L.C.); 4 Department of Electrical Engineering, Ming Chi University of Technology, New Taipei City 243303, Taiwan; 5 Department of Electronic Engineering, Feng Chia University, Taichung City 40724, Taiwan; tsungychen@fcu.edu.tw; 6 Department of Information Management, Chung Yuan Christian University, Taoyuan City 320317, Taiwan; 7 Department of Microelectronics, College of Physics and Information Engineering, Fuzhou University, Fuzhou 350108, China; eetommy@fzu.edu.cn; 8 Department of Electrical Engineering, National Cheng Kung University, Tainan City 701401, Taiwan; wctu@gs.ncku.edu.tw; 9 Ateneo Laboratory for Intelligent Visual Environments, Department of Information Systems and Computer Science, Ateneo de Manila University, Quezon City 1108, Philippines; pabu@ateneo.edu

**Keywords:** bitewing radiography, caries under dental restoration and dental braces, convolutional neural network, image enhancement, tooth segmentation, YOLOv8

## Abstract

In the dentistry field, dental caries is a common issue affecting all age groups. The presence of dental braces and dental restoration makes the detection of caries more challenging. Traditionally, dentists rely on visual examinations to diagnose caries under restoration and dental braces, which can be prone to errors and are time-consuming. This study proposes an innovative deep learning and image processing-based approach for automated caries detection under restoration and dental braces, aiming to reduce the clinical burden on dental practitioners. The contributions of this research are summarized as follows: (1) YOLOv8 was employed to detect individual teeth in bitewing radiographs, and a rotation-aware segmentation method was introduced to handle angular variations in BW. The method achieved a sensitivity of 99.40% and a recall of 98.5%. (2) Using the original unprocessed images, AlexNet achieved an accuracy of 95.83% for detecting caries under restoration and dental braces. By incorporating the image processing techniques developed in this study, the accuracy of Inception-v3 improved to a maximum of 99.17%, representing a 3.34% increase over the baseline. (3) In clinical evaluation scenarios, the proposed AlexNet-based model achieved a specificity of 99.94% for non-caries cases and a precision of 99.99% for detecting caries under restoration and dental braces. All datasets used in this study were obtained with IRB approval (certificate number: 02002030B0). A total of 505 bitewing radiographs were collected from Chang Gung Memorial Hospital in Taoyuan, Taiwan. Patients with a history of the human immunodeficiency virus (HIV) were excluded from the dataset. The proposed system effectively identifies caries under restoration and dental braces, strengthens the dentist–patient relationship, and reduces dentist time during clinical consultations.

## 1. Introduction

As technology continues to advance at a rapid pace, artificial intelligence (AI) is becoming more deeply embedded in numerous industries, with healthcare standing out as a key area of impact. AI is making significant strides in areas such as heart disease [1], cancer [2,3], and diabetes [4]. Its applications span from aiding in diagnostics to planning treatments and developing personalized medical plans, underscoring its tremendous potential to enhance the efficiency and accuracy of medical services. In dental medical diagnostics, AI has already demonstrated its transformative potential. Leveraging machine learning algorithms, AI can process extensive amounts of medical imagery, including X-rays [5], CT [6], and MRI scans [7], enabling doctors to diagnose diseases more accurately.

**Figure 1:**
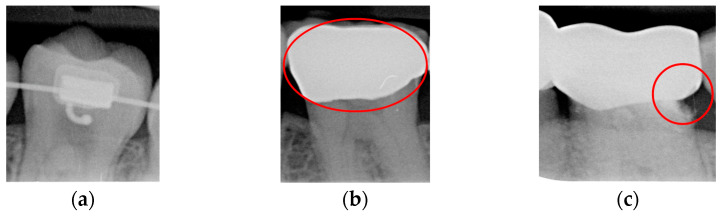
BW images with disease. (**a**) Dental braces. (**b**) The red circle represents the restoration. (**c**) The gap in the red circle indicates dental caries under the restoration.

Caries are a prevalent issue in dental healthcare, affecting nearly all adults and 60–90% of children, posing a significant public health challenge, especially with dental braces or dental restorations [8,9]. Traditional dental examinations relying on visual inspection or radiographic images [10,11] can be subjective and time-consuming. Related studies have utilized auxiliary software for oral examinations, such as methods of geometric alignment to compare noise levels in subtraction images [12], jawbone regeneration [13], and corticalization measurement [14]. With the rise of AI, automated caries detection using image processing and deep learning technologies has gained increasing attention [15]. Deep learning techniques such as convolutional neural networks (CNNs) have shown significant performance in medical image classification by leveraging large-scale annotated datasets [16,17]. In dentistry, CNNs have been applied to detect apical lesions, offering objective interpretation and reducing diagnostic time [18]. Bitewing radiographs (BWs) are commonly used to identify caries and periodontal conditions. Tooth region extraction from BWs can be performed using filtering, binarization, and projection methods [19,20,21,22]. The YOLO object detection algorithm enables real-time localization with high accuracy and speed [23,24]. This study uses YOLO to detect caries under restorations and dental braces, as illustrated in Figure 1.

Three primary methods are commonly used to diagnose dental caries: digital radiography, simulated radiography, and 3D imaging techniques such as CBCT. For example, Baffi et al. [10] reviewed 77 studies involving 15,518 tooth surfaces, with 63% showing enamel caries. Lee et al. [25] applied a U-Net-based CNN for early caries detection, achieving an accuracy of 63.29% and a recall of 65.02%. Dashti et al. [26] used deep learning on 2D radiographs and achieved an average precision of 85.9%. In addition to CNN-based methods, image enhancement techniques such as noise reduction, contrast adjustment [27], intensity value mapping [28], and histogram equalization [29] have been widely adopted to improve lesion visibility and classification performance. Well-known CNN models like AlexNet, GoogLeNet, and MobileNet have also been used for training and evaluating datasets containing secondary caries and healthy teeth, allowing for comparisons of model performance and accuracy.

Despite numerous studies employing AI-assisted methods for detecting dental caries, two key limitations remain. First, the accuracy of most existing models typically ranges between 88% and 93%, indicating a persistent risk of misclassification. Second, these studies often exclude cases involving caries under dental restorations and orthodontic braces, which limit their applicability in more complex clinical scenarios. Thus, we employ rotation-aware segmentation methods to address the various BW tilt angles to detect dental caries, ensuring that the most suitable segment angle is used for each BW and maintaining high detection accuracy despite variations in BW imaging angle. Moreover, an ablation experiment was conducted to analyze the impact of various image enhancement techniques on model performance. The proposed system was also benchmarked against recent state-of-the-art studies to evaluate its precision in detecting caries under dental braces and dental restoration. This study aims to focus specifically on detecting dental caries under dental braces and dental restorations. The proposed system is designed to assist clinicians in interpreting BW images and identifying caries under dental restorations and braces by leveraging deep learning and image processing techniques. The goal is to develop an AI-assisted diagnostic tool that reduces the diagnostic burden on dental professionals, enhances early detection accuracy, and improves clinical efficiency in real-world dental practice.

## 2. Materials and Methods

**Figure 2:**
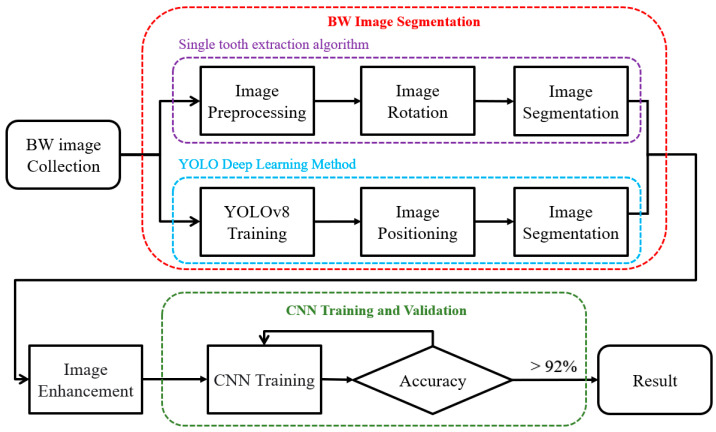
Caries under dental brace and dental restoration detection flow chart.

This study aims to develop an automated system to help dentists quickly detect caries under dental restoration and dental brace. However, the diverse shapes and orientations of teeth in BWs present significant challenges for accurate individual tooth assessment. Thus, we first locate and segment each tooth in the BW by YOLO. At the same time, we implemented the proposed rotation-aware segmentation on the BW and evaluated its performance compared with YOLO-based detection. Subsequently, we applied image processing algorithms and conducted an ablation experiment to optimize caries detection under dental restorations and orthodontic braces. These experiments enabled the model to achieve its highest detection precision by effectively isolating the target regions and minimizing background interference with CNN training. The overall flow chart is shown in Figure 2.

### 2.1. BW Image Dataset Collection

The dataset used in this study was provided by Chang Gung Memorial Hospital, Taoyuan, Taiwan. It was approved by the Institutional Review Board (IRB) of Chang Gung Medical Foundation (IRB number: 02002030B0). BW image and corresponding ground truth annotations were collected by three oral specialists, each with over five years of clinical experience. Each expert independently annotated the presence of caries under restorations and dental braces on the BW images using the LabelImg tool version 1.7.0. The annotation process was conducted without mutual influence among annotators. Final labels for each BW image were determined by majority voting to ensure annotation reliability. Patients with a history of the human immunodeficiency virus (HIV) were excluded from the dataset. All eligible BW images collected during the study period were included in the dataset to maximize sample size and ensure the generalizability of clinical diagnosis.

Model training, testing, and validation were supervised by senior researchers with extensive experience. A blinded protocol was implemented during the validation and testing stages to eliminate operator bias. Specifically, the operator conducting model evaluation was unaware of whether the BW images contained teeth affected by caries under restorations or dental braces, ensuring objective assessment. The BW dataset contained 505 images, and the single-tooth dataset included 440 images. For the tooth localization task using YOLO, 84 BW images were reserved as a validation set, while the remaining images were split into training and test sets in an 8:2 ratio. For the CNN-based classification task detecting the presence of caries under restorations and dental braces, 40 single-tooth images were reserved for validation, and the remaining images were divided into training and test sets using a 7:3 ratio.

### 2.2. BW Image Segmentation

This subsection describes our two image segmentation methods. The first method is rotation-aware segmentation, which extracts single teeth by finding the optimal rotation angle of the BW slice and segmenting based on horizontal and lead hammer lines. The second method uses the YOLO deep learning technique to determine tooth coordinates and segment teeth accordingly. These techniques allow for subsequent image enhancement and CNN training, improving the model’s ability to localize and classify caries under complex conditions.

- Single-tooth extraction algorithm

**Figure 3:**
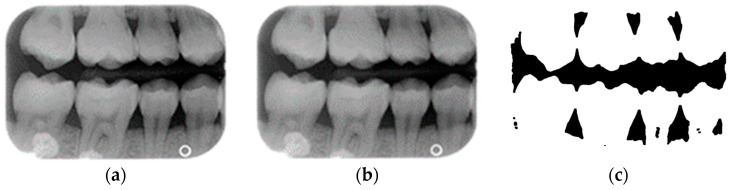
BW image preprocessing. (**a**) Original BW. (**b**) Gaussian filter. (**c**) Horizontal erosion after binarization.

A complete BW varies due to factors such as angle, exposure size, the number of teeth, and interproximal spacing. Using fixed parameters and thresholds can lead to misjudgments and low segmentation efficiency. To enhance flexibility and operability, the algorithm uses adaptive thresholds tailored to each BW based on brightness, size, and the number of teeth. Each BW is pre-processed before segmentation due to variations in mouth shape, tooth shape, and imaging angle. This study first applies to a gaussian high-pass filter to eliminate noise, reducing segmentation errors. Next, the images undergo binarization and erosion techniques to clarify background contours, making them easier to distinguish, as illustrated in Figure 3.

**Figure 4:**
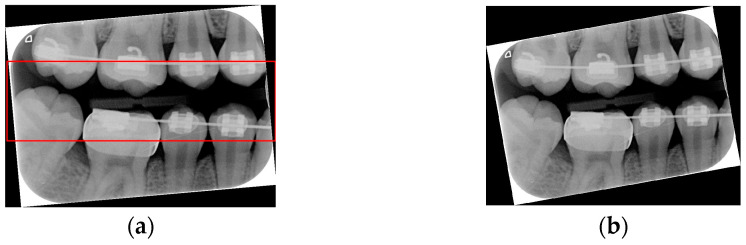
Horizontal projection of the rotated image. (**a**) BW rotated +5 degrees; (**b**) BW rotated +10 degrees.

Due to angular issues in a BW, horizontal and vertical lines may not fully separate the teeth. This study addresses this by rotating and binarizing images multiple times to enhance the contrast between teeth and gaps. High-contrast images allow for accurate identification of tooth gaps through pixel horizontal projection as shown in Figure 4a. The image is divided horizontally into three parts, masking the upper and lower sections to focus on the middle, like the upper and lower sides of the red box in Figure 4b are masked. The valleys of the projection line in this region are identified as the x-minimum value, and the y-coordinate of the valley represents the vertical height separating the upper and lower rows of teeth after rotation. Additionally, during each rotation, a projection is made to identify the trough position in the middle of the image. The trough values (x-minimum) at each angle are compared to determining the optimal rotation angle for horizontal segmentation. Initially, the image is rotated within a range of plus or minus 15 degrees, in increments of 5 degrees. By comparing the trough values at each angle, the most suitable rotation angle for horizontal cutting is identified, as shown in Figure 4b.

**Figure 5:**
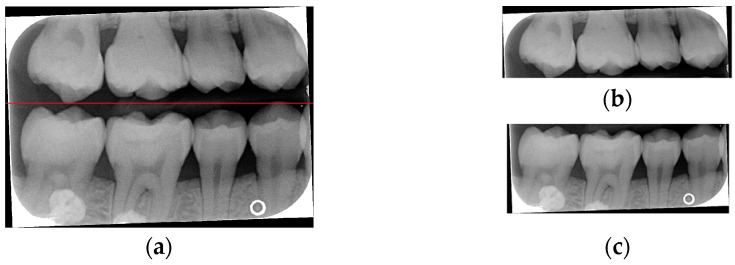
Segmentation of the upper and lower rows of teeth of the BW. (**a**) Horizontal line drawing of the lowest pixel coordinates. (**b**) Upper row of teeth. (**c**) Lower row of teeth.

According to Table 1. After performing small-angle rotations and comparing the trough values at each angle, it was determined that the lowest trough value (x = 36) occurs at a rotation of 11 degrees, which is lower than the trough value (x = 40) obtained at the initial rotation of 10 degrees. Therefore, it can be concluded that a positive 11 degrees is the most suitable rotation angle for this BW, which is more favorable for subsequent horizontal segmentation. If a smaller rotation angle is used from the beginning to find a suitable angle, multiple calculations will be required within the same range of angles. However, by gradually rotating the image in two steps, one large angle (5 degrees) and one small angle (1 degree) to obtain the most suitable rotation angle, we achieve the same result and find out the suitable angle more quickly. After rotating the image of each BW to a suitable angle, the height of the trough (y-value) is found. The height of the plumb coordinates of the troughs are found and the horizontal line separating the upper and lower jaws is plotted using the height of these coordinates. This allows the entire BW to be divided into upper and lower rows of teeth; the specific segmentation result is shown in Figure 5.

**Figure 6:**
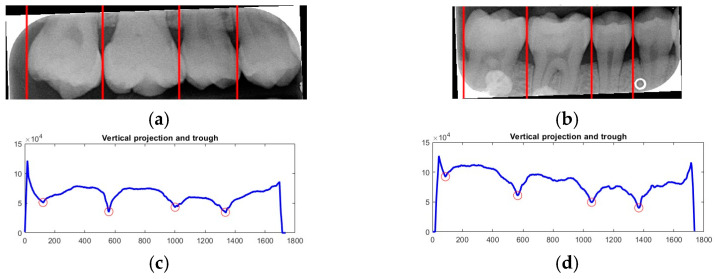
Single-tooth segmentation technology. (**a**) Upper teeth. (**b**) Lower teeth. (**c**) Upper teeth coordinates. (**d**) Lower row teeth coordinate.

**Figure 7:**
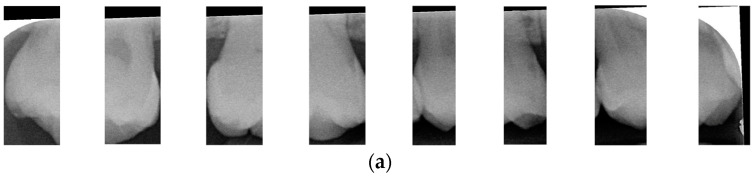

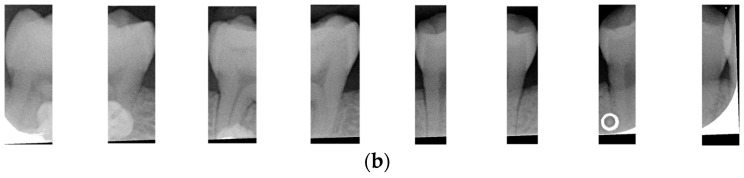
Half-a-tooth training image. (**a**) The upper teeth (**b**) The lower teeth.

After dividing the BW into upper and lower rows of teeth, each tooth is segmented individually. Vertical projection and vertical erosion are used to find the troughs (y-minimum) of the adjacent waveforms, identifying the gaps between teeth to separate each one. The number of vertical lines required varies with the number of teeth in each row. If the number of teeth is *n*, then *n − 1* vertical lines are needed for complete segmentation. These *n − 1* lines correspond to the number of troughs found in the vertical projection of the waveform. The x-coordinates of these troughs are returned to the original image, where vertical lines are drawn to isolate the teeth. The peaks and valleys are marked with red circles in Figure 6a,b, which is shown in Figure 6c,d. Because secondary caries mainly occurs on both sides of the teeth, and since each complete tooth has both a left and right half, this increases the complexity during training and judgment, resulting in poor training outcomes. Therefore, each tooth image is further divided into left and right halves, as shown in Figure 7. This approach reduced the complexity of the data and doubled the training dataset, providing more data for training.

B. YOLO Deep Learning Method

**Figure 8:**
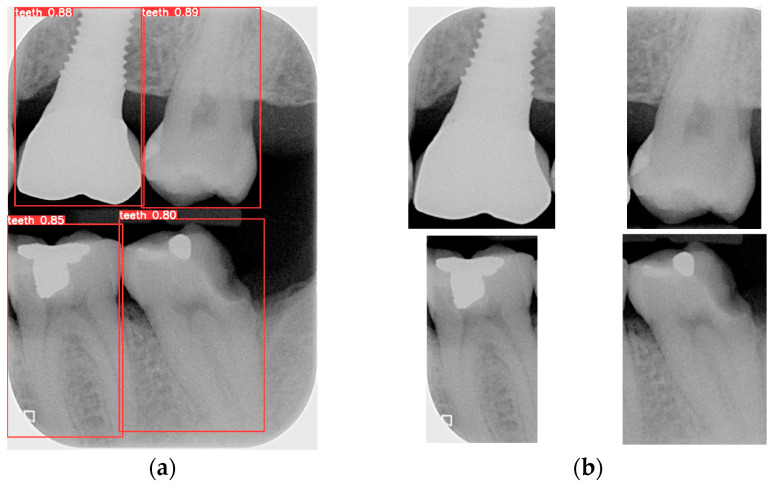
The BW is segmented after single-tooth marking. (**a**) The result of the original image after the judgment. (**b**) Segmentation results.

Object detection has been a challenging task in computer vision and deep learning. Traditional methods often require multiple steps, including region extraction, feature computation, and classification, leading to slow processing speeds and high complexity. However, recent advancements in deep learning have led to significant progress in object detection. YOLO achieves excellent accuracy and significantly outperforms traditional methods in image processing speed. Its uniqueness lies in detecting and locating objects in the entire image at once, without the need for excessive computation. YOLO is used to locate the teeth by finding the coordinates of each tooth in the BW. The BW is segmented according to these coordinates to produce an image of each individual tooth. Training the YOLO model requires a large amount of data for training and validation, with each piece of data distinguished from the target. The trained model is then applied to the entire database of BWs, identifying and labeling the position of each tooth. The BW is segmented to obtain individual tooth images after determining the coordinates of each tooth. Subsequently, the length and width data of the four teeth in the BW are used to segment each tooth, which is shown in Figure 8.

### 2.3. Image Enhancement

**Figure 9:**
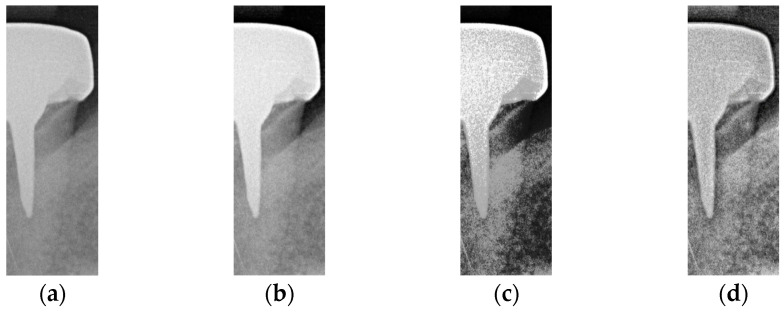
Image enhancement result. (**a**) Original. (**b**) Intensity value mapping. (**c**) Histogram equalization. (**d**) Adaptive histogram equalization.

This subsection aims to make symptomatic conditions more apparent, thereby making the images more suitable for CNN training and analysis. In a BW, tooth decay appears as black gaps, teeth appear as grayish-white, and dental restorations appear as bright white. The enhancement process focuses on increasing the contrast between black, gray, and white, particularly at the junctions of dental restorations, teeth, and cavities (black background). Non-smooth lines at these junctions indicate the presence of caries. Segmented images may lack sufficient color contrast or display subtle symptoms, which can hinder the CNN model’s ability to train and discriminate effectively. To address this, image enhancement techniques are employed to improve symptom visibility by increasing contrast. Histogram equalization (HISTEQ) is used to enhance dark and bright areas and increase overall contrast, highlighting symptom locations before CNN model training. Additionally, intensity value mapping (IAM) and adaptive histogram equalization (AHE) are applied to further enhance image quality, as illustrated in Figure 9.

**Figure 10:**
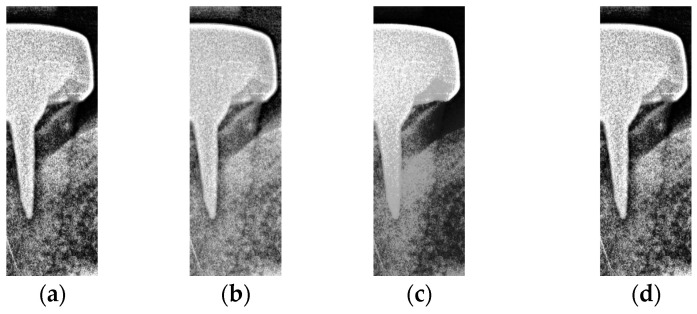
Interaction enhancement. (**a**) HISTEQ add AHE, (**b**) AHE add IAM, (**c**) IAM add HISTEQ, and (**d**) IAM, HISTEQ, and AHE.

After the above three types of symptom enhancement, it is found that the symptomatic part is not particularly noticeable. The white color of the dental restoration, the off-white color of the teeth and gingiva, and the black color of the background are not in sharp contrast. The edges of the color blocks are blurred. This may make it difficult for the CNN model to recognize the symptoms. Therefore, this study uses the above three types of reinforcement to enhance the training accuracy through the interaction enhancement model. For example, HISTEQ and AHE can increase the contrast in the image and make it easier to detect secondary caries in the image. The result of the interaction enhancement is shown in Figure 10.

### 2.4. CNN Training and Validation

Various CNN models were employed for image classification within the domain of deep learning. Using AlexNet as a representative example, Table 2 illustrates the architecture of each layer with the AlexNet model. During the training phase, each image in the validation set was individually verified to calculate the average validation accuracy. The CNN model was trained using these classified datasets, with the input image size configured to 227 × 227 × 3. This setup allowed for a consistent and standardized input size for the model. The design of the model involved modifying the last three layers, fully connected, softmax, and classification layers, and replacing them with fully connected layers specifically configured to classify the images into two categories, corresponding to the primary classes being analyzed. After the deep learning model was trained, images from the test set were randomly input into the model to assess its performance. The model classified these images based on the features it learned during the initial training phase. A confusion matrix was then generated to analyze the classification results, providing a detailed breakdown of the model’s accuracy and performance. This matrix allowed for a clear visualization of how well the model distinguished between the different classes, highlighting areas of strength and potential improvement. This systematic procedure comprehensively assessed the CNN’s performance in classifying BWs.

#### Hyperparameter Adjustment

In the training stage, each parameter represents different meanings, such as the number of layers in the neural network, the loss function, the size of the convolution kernel, and the learning rate. This study describes three modified parameters, including the initial learning rate, max epoch, and mini-batch size. Detailed hyperparameter values are listed in Table 3. The experiments were conducted on a hardware platform equipped with an Apple M1 processor (8-core CPU + 8-core GPU) operating at 3.2 GHz and 16 GB of DRAM. The software environment included MATLAB R2023a and Deep Network Designer version 14.6.

## 3. Results

This section presents the results of the YOLOv8 model and the rotation-aware single-tooth segmentation algorithm in localizing and accurately segmenting individual teeth from a BW. Both methods are evaluated to determine their effectiveness in handling image variability and ensuring reliable segmentation performance. In addition, the CNN model training outcomes are also reported. Various image enhancement techniques were applied to the segmented tooth images to investigate the influence of preprocessing. An ablation experiment was conducted to assess the impact of these enhancement methods on the performance of the CNN in detecting caries under dental restorations and dental braces.

### 3.1. Tooth Localization and Segmentation

**Figure 11:**
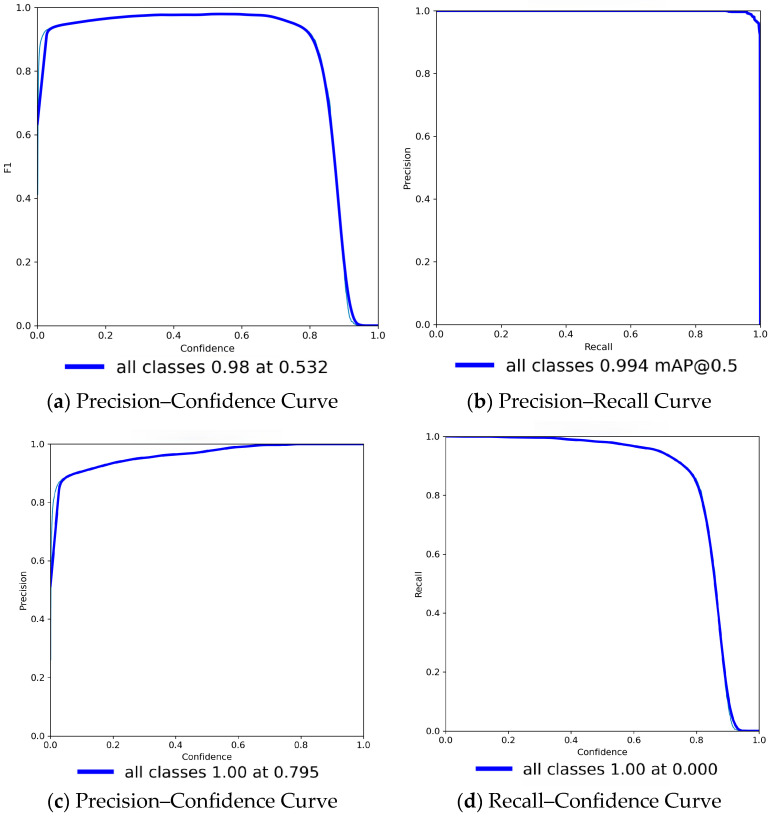
Training result data and data chart. (**a**) Confidence curve. (**b**) Precision–recall curve. (**c**) Precision–confidence curve. (**d**) Recall–confidence curve.

YOLOv8 was used as the object detection model for BWs to detect single-tooth images. The training results are illustrated in Figure 11a–d, and a comparison of these results with other methods is shown in Table 4. YOLOv8 outperforms other versions of YOLO in terms of precision, recall, and mean average precision (mAP). This study achieved a precision of 99.40%, a recall of 98.50%, and a mAP of 99.40%. Moreover, although the overall detection performance had a mAP of 0.994, the precision–recall curve slightly declined near the highest recall range. This may be attributed to a minor imbalance between the two target classes or varying recall sensitivity. The dataset consisted of two categories, and even a slight difference in sample distribution or annotation consistency could lead to observable variations in the curve. The formulas for calculating accuracy, precision, recall, and mAP are shown in Equations (1)–(5), and TP is a true positive, FP is a false positive, TN is a true negative, and FN is a false negative.

(1) $$Accuracy=\frac{TP+TN}{TP+FP+TN+FN}$$

(2) $$Precision=\frac{TP}{TP+FP}$$

(3) $$Recall=\frac{TP}{TP+FN}$$

(4) $$mAP=\frac{1}{N}\sum_{i=1}^{N}AP_{i},\;\mathrm{where}\;AP_{i}\;\mathrm{is}\;\mathrm{the}\;\mathrm{average}\;\mathrm{precision}$$

**Figure 12:**
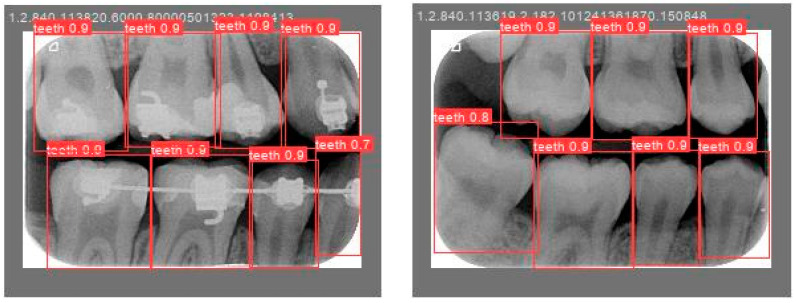
Validation results.

The validation results are shown in Figure 12. The accuracy of the judgment of a single tooth in each BW image is within the range of 80% to 90%. This high level of accuracy demonstrates that automated tools can be trusted to process large volumes of image data without requiring extensive time for individually marking the position of each tooth. Furthermore, we compared the YOLO-based method and the rotation-aware single-tooth segmentation algorithm developed in this study. As shown in Table 5, the segmentation average accuracy (ACC) of YOLOv8 is comparable to that of our proposed algorithm. However, the proposed method demonstrates a faster inference time (IT) for individual tooth segmentation and effectively addresses errors caused by variations in image tilt angles. A paired t-test was performed between YOLO and the proposed model on 40 validation images. The *p*-value of 0.013 indicates a meaningful and statistically supported improvement in performance. The paired *t*-test is shown in (5), where X_i_ and Y_i_ represent the two Intersection over Union (IoU) values of the i-th paired data. The difference d_i_ is calculated by subtracting Y_i_ from X_i_, and N denotes the total number of paired samples.

(5) $$p\text{-}value=\frac{\sum_{i=1}^{N}{(X}_{i}-Y_{i})/N}{\sqrt{\frac{1}{N(N-1)}\sum_{i=1}^{N}{{[(X}_{i}-Y_{i})-\bar{d}]}^{2}}},\;\mathrm{where}\;\bar{d}=\frac{1}{N}\sum_{i=1}^{N}d_{i}$$

### 3.2. CNN Results

**Figure 13:**
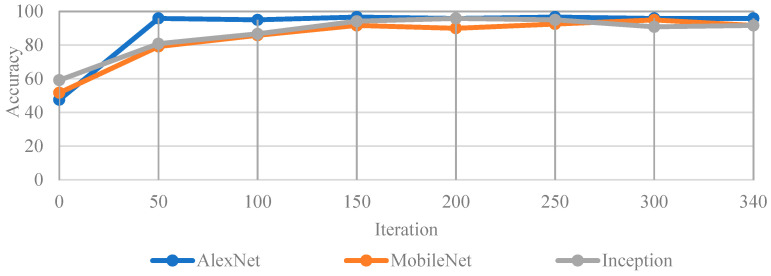
The accuracy of the original images in the test set.

In terms of CNN model accuracy, this study used the validation set for evaluation. The predictions obtained from the CNN model are compared with the correct categories of the images to obtain accuracy. The detailed training process diagram of the images without enhancement is shown in Figure 13. To evaluate the performance and accuracy of different CNN models, metrics such as precision and recall are used. The confusion matrix of the AlexNet classification model on the test dataset shows that 62 images with caries under restoration and braces (CuRB) were correctly classified, while one CuRB image was misclassified as non-caries under restoration and dental braces (N-CuRB). Additionally, 56 N-CuRB images were correctly identified, with only one misclassified as CuRB. These results indicate that the model achieved high classification accuracy with minimal false positives and false negatives in Table 6.

Table 7 presents the classification results of caries under restorations and dental braces during individual validation. BW images were preprocessed and enhanced before being classified by the CNN. The predicted results were then mapped back to the BW image to preserve clinical interpretability and ensure the model’s applicability in real-world settings. These evaluation results were produced using different CNN models: AlexNet, MobileNet, and Inception_v3. The table compares the models’ performance in identifying N-CuRB teeth and CuRB. For the N-CuRB group, our approach achieved remarkably high accuracy, with AlexNet reaching 99.94%, MobileNet reaching 92.18%, and Inception reaching 69.97%, all outperforming the corresponding accuracies in [32], which were 98.83%, 72.12%, and 52.66%. In the CuRB group, our method showed even greater improvements. AlexNet achieved 99.99%, MobileNet achieved 99.74%, and Inception achieved 94.95%, which are significantly higher than the accuracies reported in [32] (79.63%, 83.63%, and 68.09%, respectively). These results confirm the robustness of our approach in accurately classifying challenging cases involving caries beneath restorations and orthodontic appliances. Additionally, Table 8 presents a comparison of ablation experiment results after applying various image enhancement techniques. The data demonstrate the effectiveness of different CNN models and enhancement methods in improving the accuracy of dental image classification. AlexNet achieved the highest accuracy in identifying disease, with significant improvements observed after image enhancement using methods such as AHE, HISTEQ, and IAM.

**Figure 14:**
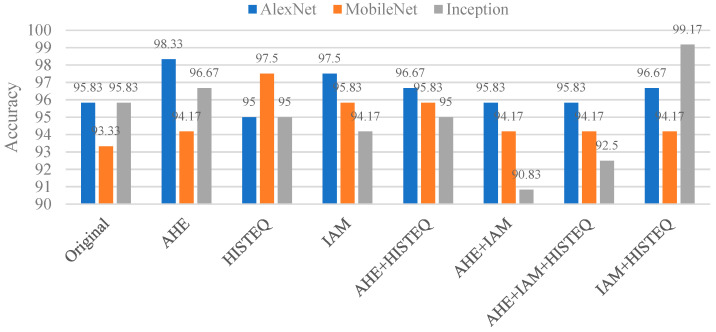
Comparison with different image enhancement methods.

Figure 14 illustrates the impact of various image enhancement techniques on the classification accuracy of three CNN architectures with AlexNet, MobileNet, and Inception for detecting caries under restoration and dental braces. Among all models and enhancement strategies, the highest overall accuracy (99.17%) was achieved by Inception using the IAM + HISTEQ combination, highlighting the effectiveness of combining intensity adjustment and histogram equalization in enhancing lesion visibility. AlexNet performed best (98.33%) when using AHE alone, while MobileNet achieved its highest accuracy (97.5%) with the HISTEQ technique. Compared to the original, unenhanced images, AlexNet, MobileNet, and Inception achieved 95.83%, 93.33%, and 95.83%; the enhancement techniques generally improved accuracy across all models. However, excessive combinations such as AHE + IAM and AHE + IAM + HISTEQ led to decreased performance, especially for Inception (down to 90.83%), likely due to overprocessing and feature distortion. These results suggest that appropriate image preprocessing plays a critical role in improving the diagnostic performance of CNN models and that the selection of enhancement methods should be tailored to the model architecture to achieve optimal outcomes in caries under restoration and dental brace detection.

Table 9 compares the accuracy between the method proposed in this study and the technique used in [32] for detecting individual teeth in a BW. In addition, we employed an external validation dataset [33] for the classification of caries under restoration and dental braces in the BW image. This open-source dataset contains 2810 BW images and was annotated by eight dentists (five senior and three junior practitioners) using rectangular bounding boxes to label carious lesions. The performance and generalizability of the proposed method were evaluated through a comparative analysis with the masking technique introduced in [32], which reduces interference from adjacent teeth without rotating the BW image. Classification accuracy was assessed using three convolutional neural network (CNN) models—AlexNet, MobileNet, and Inception_v3. Our method performed better than [32], achieving an accuracy of 99.17% with Inception_v3, compared to the reported accuracy of 80.00% using MobileNet in [32]. In addition, we used an external validation open-source dataset [33] to evaluate our model’s generalizability. Without image enhancement, the classification accuracies were 92.01%, 93.45%, and 93.58% for AlexNet, MobileNet, and Inception_v3. After enhancement, performance improved to 96.42%, 97.11%, and 97.89%. These results demonstrate the effectiveness and robustness of the proposed enhancement strategy in improving caries classification accuracy across both internal and external datasets.

## 4. Discussion

This study uses deep learning techniques to detect whether individual teeth in a BW affected by dental restorations and dental braces exhibit signs of caries. To enhance model performance, image processing and enhancement techniques are incorporated to improve the training outcomes of the deep learning models. This system is primarily designed to support dental professionals in clinical diagnosis and aims to serve as a diagnostic aid, especially for senior dentists in learning to identify carious lesions. Moreover, this study addresses a significant gap in current research, where caries under dental restorations and dental braces have often been excluded from diagnostic models. Compared to previous studies, our experiment results better detect caries beneath restorations and around braces. For instance, Ayhan et al. [34] developed a CNN model using U-Net for caries detection on bitewing radiographs, achieving a precision of 65.1% and a recall of 72.7%. In contrast, our model achieved higher precision and recall rates, indicating improved diagnostic accuracy in complex restorations and orthodontic appliance cases. Furthermore, Pérez de Frutos et al. [35] utilized deep learning methods for detecting proximal caries lesions in BW images, emphasizing the potential of AI in enhancing diagnostic capabilities. Our inclusion of images with restorations and orthodontic appliances in the dataset addresses the limitations noted in earlier research, where such complexities were often excluded. Additionally, our model’s performance metrics surpass those reported in prior studies utilizing similar deep learning architectures for caries detection, indicating a significant advancement in diagnostic accuracy. This is particularly evident when compared to the work of Ayhan et al. [36], who implemented a deep learning approach for caries detection and segmentation on bitewing radiographs, which achieves a precision of 93.4% and a recall of 83.4%, and in our YOLOv8 detection can reach 99.4% and 98.5%, demonstrating that our result is better than the state-of-the-art research. Overall, the contributions and innovations in this study are as follows:

1. We evaluated two segmentation techniques for BWs, including the state-of-the-art YOLOv8 model and our innovative rotation-aware single-tooth segmentation algorithm, which effectively compensates for errors caused by angular variations in BWs. While both methods achieved comparable segmentation accuracy (96–98%), our proposed algorithm showed a faster inference time, at least twice as fast as YOLOv8.
2. We compared our deep learning model with recent BW-based single-tooth detection studies [30,31]. We observed improvements, with precision increasing by up to 13.25% and recall by 12.55%. The proposed method achieved a maximum precision of 99.40% and a recall of 98.50% in detecting the targeted lesions.
3. In detecting caries under restoration and dental braces, we applied various image enhancement techniques and conducted ablation studies to verify their effectiveness. The better-performing model is Inception-v3, which achieved an accuracy of 99.17%, representing a 3.34% improvement over the baseline without enhancement. Compared with a recent method in [32], our system showed a 26.36% improvement in lesion detection.
4. To evaluate clinical applicability, we tested the system on external datasets not used for training or validation. The system achieved over 90% accuracy in identifying caries under dental restorations and dental braces cases, demonstrating its robustness and stability in practical diagnostic scenarios.

Despite the significant technical progress made in this study, several limitations remain. First, the limited size of the original dataset may affect the model’s generalization capability. Although data augmentation techniques were employed to alleviate sample insufficiency, further validation using large-scale and diverse datasets is necessary. We addressed this concern by using an external open-source dataset [33] that was entirely separate from the training and validation data to evaluate the model’s robustness. In addition, we plan to conduct prospective real-world validation in collaboration with multiple medical institutions, aiming to expand our radiographic database and improve the clinical applicability and stability of the proposed system. Second, although classic CNN architectures such as AlexNet and Inception-v3 have demonstrated strong accuracy in this study, they were primarily chosen due to their stability on moderately sized datasets and relatively low computational requirements, making them suitable for initial validation stages. However, recent studies have shown that Vision Transformer (ViT) models exhibit superior performance in medical image analysis, particularly in capturing long-range dependencies and global features, which are essential for identifying complex structures. ViT typically requires large-scale datasets to achieve high accuracy. Azad et al. [37] have shown that the effectiveness of ViT architectures in medical imaging tasks is significantly influenced by the availability of extensive training data. In future work, we will collect more BW datasets and combine ViT and assess their potential benefits in enhancing model performance and generalization capability.

Third, while effective, the current image enhancement strategy retains substantial background information during lesion detection, especially when identifying implants, where non-lesion areas may remain overly prominent. Future work will explore alternative enhancement and preprocessing techniques to suppress irrelevant backgrounds better and emphasize pathological regions. Fourth, this study did not consider the phenomenon of cervical burnout, which can mimic carious lesions on BW images and potentially lead to false-positive detections. In future work, we will explore artifact-reduction techniques and model adaptations to distinguish true lesions from cervical burnout. This study did not specifically address the issue of radiolucent restorative materials, such as certain composite resins, which may mimic carious lesions on radiographs. These materials can appear as radiolucent and may be mistakenly identified as caries, posing a risk of false-positive diagnoses. In future work, we plan to investigate methods to differentiate true caries from radiolucent artifacts. Additionally, integrating the system into clinical practice requires addressing compatibility with existing dental software and meeting regulatory standards. We will work closely with practitioners to optimize usability and ensure compliance with clinical guidelines.

## 5. Conclusions

The primary objective of this study is to enable the automated and accurate diagnosis of caries under restoration and dental braces, assisting dental professionals in improving treatment efficiency. The final experimental results demonstrate that the proposed method effectively detects this specific type of lesion. This aligns with the study’s specific aim of addressing diagnostic challenges in cases where conventional image interpretation may be obscured by restoration or dental braces. In future work, we aim to collaborate with dental practitioners to validate the model in real-world clinical settings, ensuring its practicality and reliability. Clinically, the system has the potential to serve as an assistive tool that supports early detection, reduces diagnostic workload, and enhances decision-making in routine dental practice. By achieving these goals, this study seeks to advance the field of dental imaging and provide a valuable tool for the early detection and treatment of dental diseases, contributing to more intelligent and efficient dental care solutions.

## Figures and Tables

**Table 1:** BW rotation based on horizontal projection at every angle.

Angle	−15°	−10°	−5°	0°	5°	6°	7°
x coordinate	854	785	516	407	261	179	135
Angle	8°	9°	10°	11°	12°	10°	15°
x coordinate	78	42	40	36	45	40	281

**Table 2:** The input and output of the AlexNet model.

	Type	Activations
1	Image Input	227 × 227 × 3 × 1
2	2-D Convolution	55 × 55 × 96 × 1
3	ReLU	55 × 55 × 96 × 1
4	Cross Channel Normalization	55 × 55 × 96 × 1
5	2-D Max Pooling	27 × 27 × 96 × 1
6	2-D Grouped Convolution	27 × 27 × 256 × 1
7	ReLU	27 × 27 × 256 × 1
8	Cross Channel Normalization	27 × 27 × 256 × 1
9	2-D Max Pooling	13 × 13 × 256 × 1
10	2-D Grouped Convolution	13 × 13 × 384 × 1
11	ReLU	13 × 13 × 384 × 1
12	2-D Grouped Convolution	13 × 13 × 384 × 1
13	ReLU	13 × 13 × 384 × 1
14	2-D Grouped Convolution	13 × 13 × 256 × 1
15	ReLU	13 × 13 × 256 × 1
16	2-D Max Pooling	6 × 6 × 256 × 1
17	Fully Connected	1 × 1 × 4096 × 1
18	ReLU	1 × 1 × 4096 × 1
19	Dropout	1 × 1 × 4096 × 1
20	Fully Connected	1 × 1 × 4096 × 1
21	ReLU	1 × 1 × 4096 × 1
22	Dropout	1 × 1 × 4096 × 1
23	Fully Connected	1 × 1 × 2 × 1
24	Softmax	1 × 1 × 2 × 1
25	Classification Output	1 × 1 × 2 × 1

**Table 3:** Hyperparameters in the CNN model.

Hyperparameters	Value
Initial Learning Rate	0.0001
Max Epoch	20
Mini Batch Size	16
Learning Drop Period	10
Learning Rate Drop Factor	0.1

**Table 4:** Accuracy of single-tooth detection using YOLO and comparison with other research.

		Precision	Recall	mAP
This Study	YOLOv8	99.40%	98.50%	99.40%
Method in [30]	YOLOv3	86.15%	85.95%	81.43%
Method in [31]	YOLO v5S	61.90%	70.90%	64.90%
	YOLO v5M	71.20%	70.80%	70.50%
	YOLO v5L	64.30%	68.10%	68.20%

**Table 5:** Comparison between YOLOv8 and rotation-aware single-tooth segmentation algorithm.

Method	Metrics	Angle				
		−10°	−5°	0°	5°	10°
YOLOv8	ACC	96.23	97.88	98.19	97.11	97.45
	IT	3.25 s	3.34 s	3.56 s	3.18 s	3.47 s
Algorithm	ACC	97.58	97.10	97.84	98.09	96.66
	IT	1.55 s	2.01 s	1.59 s	1.55 s	2.17 s

**Table 6:** AlexNet test dataset confusion matrix used for caries under restoration and dental braces caries (CuRB).

		Actual	
		CuRB	N-CuRB
Predicted	CuRB	62	1
	N-CuRB	1	56

**Table 7:** Comparison of clinical data and the validation image.

Ground Truth: Non-caries under restoration and dental braces (N-CuRB)	Model	This Study	Method in [32]
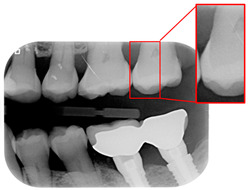	AlexNet	99.94% to be N-CuRB	98.83% to be N-CuRB
	MobileNet	92.18% to be N-CuRB	72.12% to be N-CuRB
	Inception	69.97% to be N-CuRB	52.66% to be CuRB
Ground Truth: Caries under restoration and dental braces (CuRB)	Model	This Study	Method in [32]
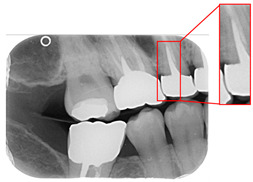	AlexNet	99.99% to be CuRB	79.63% to be CuRB
	MobileNet	99.74% to be CuRB	83.63% to be N-CuRB
	Inception	94.95% to be CuRB	68.09% to be N-CuRB

**Table 8:** Image enhancement ablation experiment with AlexNet model.

Method	Original	AHE	HISTEQ	IAM
Accuracy	95.83%	98.33%	95.00%	97.50%
Method	AHE + HISTEQ	AHE + IAM	IAM + HISTEQ	AHE + IAM + HISTEQ
Accuracy	96.67%	95.83%	96.67%	95.83%

**Table 9:** Comparison of CNN validation with open-source dataset and state-of-the-art model.

Method	AlexNet	MobileNet	Inception_v3
Before Enhancement	95.83%	93.33%	95.83%
After Enhancement	98.33%	97.50%	99.17%
External [33] Before Enhancement	92.01%	93.45%	93.58%
External [33] After Enhancement	96.42%	97.11%	97.89%
Method in [32]	77.89%	80.00%	69.47%

## Data Availability

The data used in this study are confidential and cannot be provided to any external parties. The original contributions presented in this study are included in the article. Further inquiries can be directed to the corresponding authors.
